# The Nice Young Man

**Published:** 1882-09

**Authors:** 


### THE NICE YOUNG MAN.

NOT the young gentleman who dresses with finished elegance, and sports on Broadway the killing moustache, the white kid and the cigar; who can bow with exquisite grace; whose white teeth and dark hair and self-possessed mien, would turn the head of any boarding-school girl in the city. He was a Quaker youth, as plain in person and in dress as the plainest of his class. He was the son of a man who was rich by inheritance, but losing every thing, this son, raised to the expectation of a fortune, promptly resolved to learn a trade, and apprenticed himself to a bricklayer. As soon as he became master of his calling, he made his way to New-York, and might have been seen any day on one of its Broadway buildings, at twelve dollars a week, or about four hundred a year, as he had to lose bad weather. He found a good boarding-house with a class above him, socially. How these Quakers always manage to have the best things and the best places in their sphere! There were clerks there who were receiving twelve hundred dollars a year, with this difference, he never went in debt, always paid his bills and always had money, while his companions were always "short," always in arrears, always hard run. He was never in a hurry; was always at his post before the hour ot work, and was among the very last to "knock off" at the signal stroke. He took no drives to the Park on Sundays, and always gave the negro minstrels and the theater a wide birth. After the day's work was done, he took a bath, put on his best clothing, took his tea, then made his way to that noble institution, the Free Reading-room of the Cooper Union. A specimen of manly vigor and moral beauty, he soon became "foreman," at increased wages, and, as might be expected, attracted the special notice of his employer, worth hundreds of thousands literally, with an only daughter, etc.

What was he working for? What was his ambition? To secure by his earnings the old homestead for his mother! This is being a "nice young man" in the noblest sense. Let the many youths of the country, whose fathers, so recently wealthy, are now worse than penniless, learn a useful lesson from this narration, and "go and do likewise," instead of lounging about in idleness, waiting for something to turn up

